# Photoacoustic Effect of Near-Infrared Absorbing Organic Molecules via Click Chemistry

**DOI:** 10.3390/molecules27072329

**Published:** 2022-04-04

**Authors:** Wenqing Zhu, Zongcheng Miao, Yaqin Chu, Liaoliao Li, Lei Wang, Dong Wang

**Affiliations:** 1 School of Chemical and Environmental Engineering, Anhui Polytechnic University, Wuhu 241000, China; zwq_1021@163.com (W.Z.); yaqinjucinda@163.com (Y.C.); 2 School of Materials Science and Engineering, University of Science and Technology Beijing, Beijing 100083, China; lll285687@163.com; 3 Key Laboratory of Auxiliary Chemistry and Technology for Chemical Industry, Ministry of Education, Shaanxi University of Science and Technology, Xi’an 710021, China

**Keywords:** near-infrared, photoacoustic imaging, photothermal treatment, photodynamic therapy

## Abstract

Near-infrared dyes were developed to be contrast agents due to their ability to improve the productivity of photoacoustic (PA) imaging and photothermal therapy (PTT) treatments. During the article, we described in detail the PA and PT effects of a category of organic molecules. F_4_-TCNQ could potentially cause a red-shift in the peak PA intensity. The results show that the PTT intensity of the near-infrared dyes with phenyl groups were higher than near-infrared dyes with thiophene groups. We also investigated the photodynamic treatment effect of C1b to demonstrate that these dyes are highly desirable in biochemistry. The high photoacoustic intensity of the organic molecules and the good yield of reactive oxygen species could indicate that these dyes have good potential for a wide range of imaging applications. Finally, we embedded the dye (C1b) in a liposomal hydrophobic phospholipid bilayer (C1b⊂L) to facilitate the application of hydrophobic dyes in biomedical applications, which can be absorbed by cells with good compatible and high stability for the imaging of cellular PA.

## 1. Introduction

Photoacoustic (PA) imaging has been developed as a noninvasive real-time modality to be used for biomedical imaging, with promising applications in terms of increased penetration depth and superior spatial resolution in comparison to conventional optical imaging techniques. PA imaging can effectively image the structure and function of biological tissues. PA imaging contrast agents enhance image contrast and resolution by modifying the optical and acoustic properties of local tissues, thereby significantly improving image output [1,2,3,4]. Near-infrared light-absorbing materials have acquired favor as investigational PA contrast agents. There are two reasons for this: on the one hand, native light from tissues has the lowest absorption in the near-infrared (NIR) region, and on the other hand, PA contrast agents can potentially be used in photothermal therapy (PTT) while the materials show a strong Near-infrared absorption [5]. PA imaging contrast agents with near-infrared (NIR) window absorption and high PA intensity can effectively enhance the use of PA imaging in biomedical applications. Several NIR absorbing contrast agents were reported for photoacoustic imaging and photothermal therapy treatment, for example, near-infrared dyes [6], gold-based nanoparticles [7,8,9], along with other inorganic materials [10,11]. Nevertheless, NIR absorbing low molecular weight organic dyes exhibit excellent biodegradability and potentially short-term toxicity compared to other inorganic contrast agents which are better suited for photoacoustic and photothermal contrast agents [12].

Some organic dyes have the maximal absorption in the near infrared range and are extensively applied as PA contrast agents. For example, cyanide [13], porphyrins [14] and boron dibromothiophene derivatives [3]. The discovery of new photoacoustic molecular materials has been restricted by the paucity of systematic studies on factors affecting PA effects, which has led to poor guidance in the study of molecular photoacoustic contrast agent design [4,15,16]. Lately, the chemistry of [2 + 2] cycloaddition-cycloreversion reactions between tetracyanoethylene (TCNE), 7,7,8,8-tetracyanodimethane (TCNQ) or 2,3,5,6-tetrafluoro-7,7,8,8-tetracyanodimethane (F_4_-TCNQ) and ‘electronically chaotic’ alkynes were focused on [17,18,19,20,21,22,23,24,25,26,27,28,29,30,31,32,33,34,35,36,37,38,39]. The consequent adducts, specifically those composed of F_4_-TCNQ formation, had excellent solubility and could be easily produced in high yields. It is worth noting that these adducts display intense absorption in an area of 700–900 nm that makes them very applicable as contrast agents for PAI.

The purpose of our study was to investigate the PA and PTT effectiveness of organic molecules with the clicked 7,7,8,8-tetracyanoquiodimethane (TCNQ) and 2,3,5,6-tetrafluoro-7,7,8,8-tetracyanoquiodimethane (F_4_-TCNQ) as well as modifying all kinds of functional groups in various locations. The organic molecule clicked by F_4_-TCNQ can cause a significant red shift of the PA intensity peak compared to four different molecules. In addition, the molecules system of the conjugate structure also influences the PA intensity, which could increase the molecular conjugation length and could slightly account for the increase of PA intensity. To provide additional confirmation that C1b with the maximum PA intensity can be applied to PDT treatment, ROS yield measurements were also taken and ROS release occurred for all molecules. C1b with the best PA and PT effect was inserted into the liposomal hydrophobic phospholipid bilayer (C1b⊂L) to further investigate its toxicity and cell PA effect.

## 2. Materials and Methods

**Figure 1:**
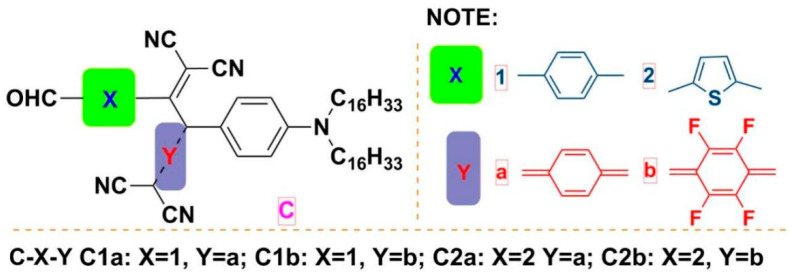
Molecular structures of compounds were named as CXY, X and Y were different modified moieties.

Our laboratory has synthesized a series of NIR absorbing molecules, as shown in Figure 1 [26,33]. C was the matrix molecular structure, and its aniline group [40] and long-chain alkyl groups could increase solubility [41] and electron cloud density. C-XY’s series were obtained by applying click reagents, such as TCNQ and F_4_-TCNQ. X and Y were the distinct groups shown in Figure 1. The spectral properties of the material are changed by the introduction of click reagents, and the click chemistry modification decreased the separation difficulties of the synthesized C-XY molecules [33].

*PA imaging in phantom:* The C-XY solution was poured into agarose tubes at a 3 × 10^−5^ mol/L concentration. The model was scanned by using Multispectral Optoacoustic Tomography (MOST) 128 in the wavelength range of 680 to 980 nm. PA intensity was acquired by averaging the pixel intensities of the same regions in images of the same laser intensity.

*The heating/cooling curves of C-XY:* The 150 mµL C-XY solution at a consistency of 3 × 10^−5^ mol/L in Tetrahydrofuran (THF) were filled into the lid of centrifuge tube which was irradiated with 400 mW power laser sapphire femtosecond laser and a 4 mm diameter mask. The wavelengths were 692 nm for C1a, 842 nm for C1b, 695 nm for C2a, and 866 nm for C2b. The temperatures were recorded by portable infrared thermometer.

*The determination of quantum yield of singlet oxygen of C-XY solution:* The C-XY molecule and the tetraphenylporphyrin (TPP) used as a reference were each soluble in THF at 3 × 10^−5^ mol/L concentration. This solution (500 µL) was then incorporated into the centrifuge tube. After that, a pre-made solution of 1,3-diphenylisobenzofuran (DPBF) dissolved in THF at the concentration of 3 × 10^−5^ mol/L (500 µL) was incorporated in a centrifuge tube. The absorbance of mixtures was determined at 410 nm using a JASCO V-570 spectrometer at intervals of 0, 10, 20, 30, 40, 50, 60, 70, 80, 90, 100 and 110 s after 650 nm laser irradiation with a laser energy density of 4.0 mW/cm^2^.

*The preparation of C1b⊂L:* L-α-phosphatidylcholine and cholesterol in the weight ratio of 4:1 were solubilized in ethanol solution. C1b solution was then added to the mentioned mixture. The obtained mixture was placed in phosphate-buffered saline (PBS) and stirred for 3 min with an ultrasonic cell disruptor, which enabled the molecules to be embedded in the liposomal hydrophobic phospholipid bilayer.

*Cell imaging in agar-based phantom:* MCF-7 cells grown in DMEM comprising 10% FBS and 1% penicillin-streptomycin were cultured for 2 h with 10 μM C1b⊂L at 37 °C and 5% CO_2_. The cells were subsequently washed three times by cold PBS, which was harvested with trypsin. Approximately 8 million cells from PBS were blended with 1% ultrapure agarose in PBS in a 1:1 ratio and they were syringed into the pores of an agarose gel phantom. Later, the pores were capped with another warm layer of agarose in a 1:1 ratio of agar powder to ultrapure water. After that, the agarose gel phantom described above were cooled at ambient temperature. By using MOST 128, PA imaging was gained at wavelengths from 680 to 980 nm.

## 3. Discussion and Results

**Figure 2:**
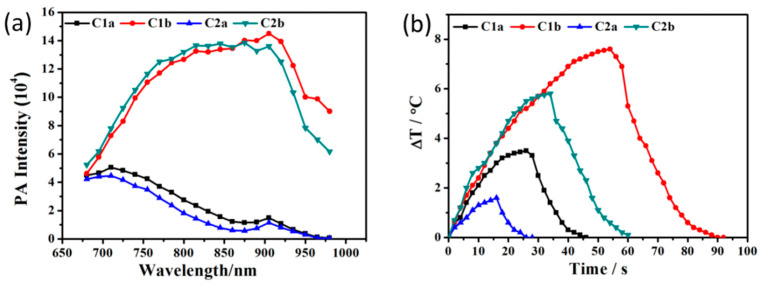

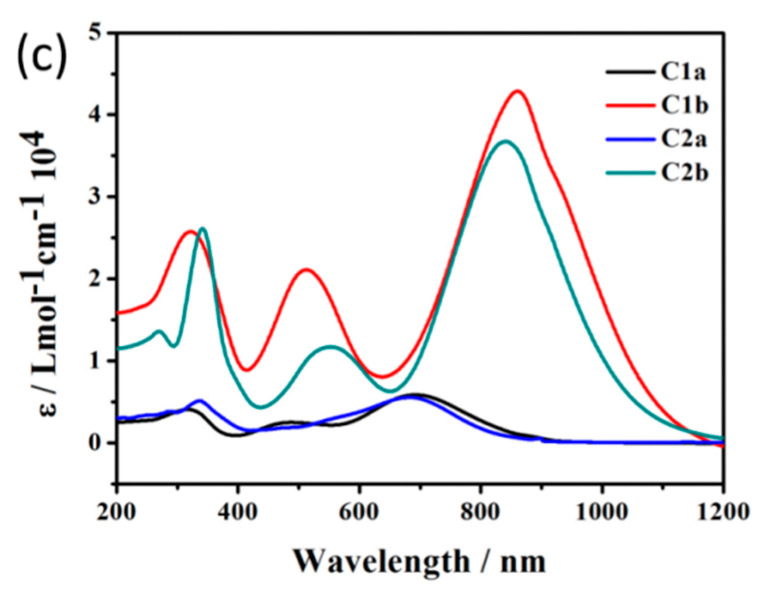
(**a**) PA intensities of C-XY materials measured for a sample concentration of 3 × 10^−5^ M in THF. (**b**) Heating/cooling curves of C-XY as function of time. (**c**) Molar extinction coefficients (ε) of C-XY measured in THF.

C1b and C2b showed good PA effects were observed as shown in Figure 2a. To obtain further insight into the PA intensity of the molecules, the extinction coefficient (ε) of the molecule was also determined. On the basis of the equation of the photothermal mechanism of the PA effect:

(1) q ∝ ΓεηF,

The thermal conversion efficiency (η) was also measured from the cooling curves shown in Figure 2b and Figure S2; where Г shows the Gruneisen parameter (dimensionless), ε indicates the optical absorption coefficient (cm^−1^), η represents the thermal conversion efficiency, as well as F is the local optical fluence (J·cm^−2^) [42]. The main parameters contributing to the PA signal are ε and η (whose values were measured by fitting the curve to the temperature with time [43]). The UV/Vis/NIR absorption (Figure 2c) were not consistent with the wavelength related PA intensity. (Figure 2a). C1b had the highest ε, so that it has the highest PA intensity. The η of the C-XY dyes shown in Table 1 was not consistent with the PA intensity at the same time. The highest η value is for C1a (55.9%), which displayed a low PA intensity. (5.1 × 10^4^), (Table 1). The outcome was not backed up by the equation of the photothermal mechanism of the PA effect. For a range of NIR dyes, the photoacoustic wave may also be associated with the electrostriction of solutions, caused by the transfer of charge from the molecule [44]. The peak PA intensities around 860 nm for both C1b and C2b which were clicked by F_4_-TCNQ. Nevertheless, clicking by TCNQ, both C1a and C2a peak at wavelengths near 690 nm. (Figure 2c). F_4_-TCNQ possessed a strong electron-absorbing group added to F, so its peaks could be shifted to long wavelengths [45]. The PA intensity and η of C1a and C1b which introduced phenyl group were greater than that of C2a and C2b with the introduction of thienyl group. (Table 1).

**Figure 3:**
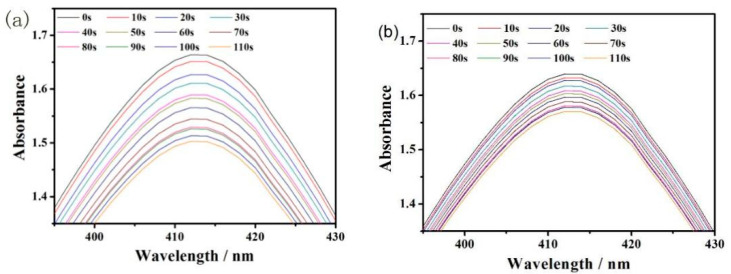
The absorbance values of TPP (**a**) and C1b (**b**) THF solution mixed with DPBF after being irradiated 650 nm laser every 10 s.

To confirm that C1b which has the highest PA intensity can as well be used as PS, the yield of ROS was investigated (Figure 3 and Figure S4). Based on the previously published method [46], this value (the yield of singlet oxygen of C-XY solution) can be determined by the formula:

(2) $$\Phi_{\Delta }^{S}=\Phi_{\Delta }^{R}\frac{K^{S}F^{R}}{K^{R}F^{S}},$$

Among them, the yield of single oxygen (Φ = 0.64) was the reference object. The marked S and R stand for the sample (C-XY) and reference (TPP (5,10,15,20-tetraphenylporphyrin)), separately. K was the linear relationship slope between the absorbance value (ΔOD) of DPBF (1,3-diphenylisobenzofuran) at 410 nm and the time of excitation T (Figure S4). F as an absorption correction factor (F = 1-10^-OD^). OD was the absorbance value of the solution at the laser light wavelength [47]. The OD of C1b was 0.24 according to Figure S4 with a K^S^ of 0.00048. The ROS yield of C1b was 24.0%, which indicates the potential use of C1b for PDT treatment.

**Figure 4:**
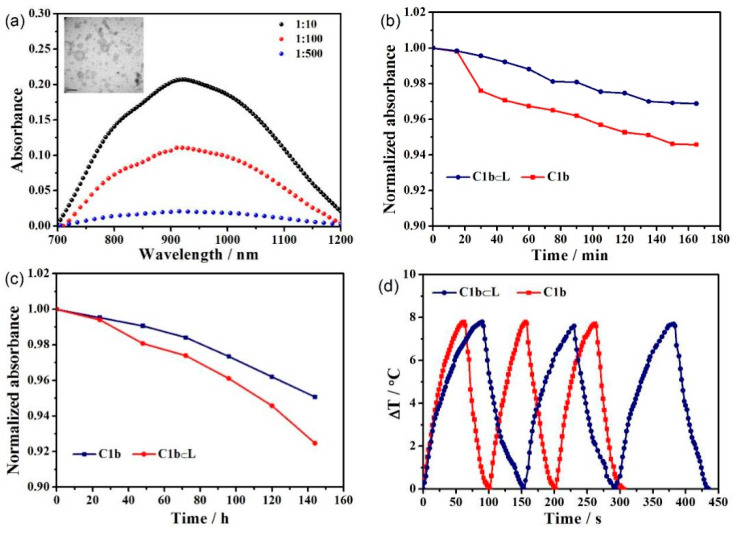
(**a**) UV/Vis/NIR absorption spectra of C1b⊂L with different ratios of C1b to liposomes in PBS and TEM images of C1b⊂L. (**b**) The UV/Vis/NIR absorption spectra were estimated after the solution of C1b and C1b⊂L illuminating in every 15 min at 860 nm. (**c**) The UV/Vis/NIR absorption spectra were estimated after the solution of C1b and C1b⊂L placing 24 h at 860 nm. (**d**) Thermal conversion efficiency of the solution of C1b and C1b⊂L was measured for continuous 3 times.

For the demonstration of further applications of a series of C-XY in PA imaging, C1b was packed in a pre-made bilayer of hydrophobic liposomes, which were extensively used as drug delivery carriers [48,49]. L-α-phosphatidylcholine and cholesterol in the weight ratio of 4:1 were solubilized in ethanol solution. Subsequently, the THF solution of C1b was incorporated to the premixed ethanol solution as described above. The obtained solution was added to phosphate-buffered saline (PBS) and agitated continuously for 1 hour. C1b molecules were perfectly inserted into the liposomal hydrophobic phospholipid bilayer by hydrophobic interaction [50]. The morphology of C1b⊂L in phosphate-buffered saline was studied using transmission electron microscopy (TEM). When the mass ratio of C1b to phospholipid was 1:10, the vesicles of C1b⊂L had a uniform structure with a dimension of 53 ± 50 nm (Figure 4a). The hydrodynamic diameter of C1b⊂L was determined using dynamic light scattering (DLS) technique with a narrow size distribution of 64 ± 17 nm (Figure S5). The C1b⊂L with a mass ratio of 1:10 showed high absorption at 860 nm, which indicated that C1b was succeeded to be mounted in the liposome (Figure 4a). In addition, we also investigated the stability of C1b mixtures and C1b⊂L in the PBS solution. After irradiation at 860 nm at 15 min intervals for 3 h, the absorption of C1b⊂L PBS solution and C1b mixed solution were still higher than 94% (Figure 4b). The absorption of C1b⊂L and C1b solutions remained above 92% after 7 days (Figure 4c), and the heating/cooling curves of C1b⊂L and C1b remained nearly constant over three consecutive times (Figure 4d). On the basis of Figure 4b–d, C1b⊂L and C1b have excellent stability under both chemical and physical conditions.

**Figure 5:**
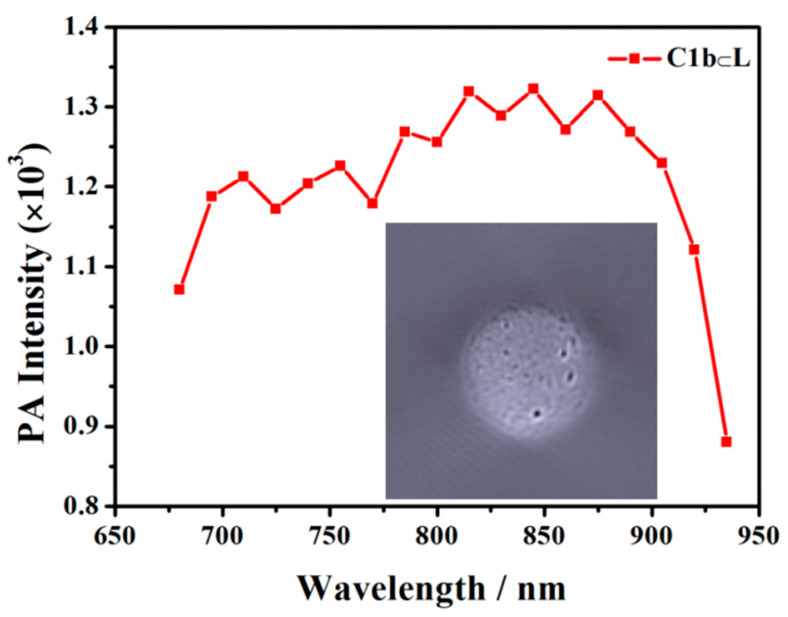
PA imaging of C1b⊂L incubated in cells and the photo of agarose gel phantom loaded with C1b⊂L incubated in cells.

Human breast cancer MCF-7 cells were used as a model cell line and the C1b⊂L served as a control agent for PA imaging in vitro. The MCF-7 cells (~107 cells) grown on culture dishes with C1b⊂L (10 mM based on C1b molecule) were incubated at 37 °C for 2 h. The treated cells in PBS and 1% ultra-pure agarose solution were mixed at a volume ratio of 1:1 and then incorporated into the holes of the pre-made agarose gel model. PA imaging was determined with MSOT 128 under 690 nm laser excitation. PA imaging was performed by recording the PA signal of C1b⊂L in the cells in the agarose gel. Since the ratio of C1b embedded in liposomes and C1b⊂L that was loaded into MCF-7 cells was not 100%, the PA intensity of C1b⊂L in cells could still achieve 1.3 × 10^4^ (Figure 5). The cellular toxicity of C1b⊂L nanoparticles was assessed by CCK-8 assay to ascertain the metabolic viability of MCF-7 cells [51]. Under our experimental conditions, there was no significant cytotoxicity detected at concentrations up to 10 mM (Figure S6).

## 4. Conclusions

In summary, a series of organic molecules with PA and PT effects were characterized with click TCNQ, F_4_-TCNQ, respectively. NIR dyes consisting of phenyl clicks has the higher PA intensities compared to NIR dyes consisting of thiophene clicks, and the introduction of the click reagent F_4_-TCNQ leads to peaks of PA intensity that are red-shifted. At the same time, C1b with optimal PA effect can generate ROS and perform PDT treatment. In addition, C1b was packed into nano-sized liposomes for further application to cells. It was demonstrated that the PA intensity was high when the liposomes remained in the cells with the molecules for 2 h, while the toxicity of the hydrophobic phospholipid bilayer (C1b⊂L) embedded in the liposomes was low, which suggests that organic molecules probably have a high potential for the detection as well as treatment of tumors in vivo.

## Figures and Tables

**Table 1:** Summary of PA intensity, ε, ΔT, τ_s_ and η of the series of C-XY.

	PA (10^4^)	ε (10^4^/mol/cm)	ΔT (°C)	τ_s_	η (%)
C1a	5.1	0.6	3.5	5.5	55.9
C1b	14.5	4.3	7.6	13.0	38.4
C2a	4.5	0.6	1.6	3.8	39.2
C2b	13.9	3.7	5.8	11.5	33.1

## Data Availability

Not applicable.

## Supplementary Materials

The following are available online https://www.mdpi.com/article/10.3390/molecules27072329/s1. Figure S1. (a) UV/Vis/NIR absorption spectra of C1b⊂L with different ratios of C1b to liposomes in PBS and TEM images of C1b⊂L. (b) The UV/Vis/NIR ab-sorption spectra were estimated after the solution of C1b and C1b⊂L illuminating in every 15 min at 860 nm. (c) The UV/Vis/NIR absorption spectra were estimated after the solution of C1b and C1b⊂L placing 24 h at 860 nm. Figure S2. Time constants for heat transfer of C-XY were acquired by ap-plying the linear time data from cooling period. Figure S3. C1a, C1b, C2a, C2b, TPP in THF with DPBF for 10 s at a time under a 650 nm light. Figure S4. Linear fit of time and ΔOD at UV absorption of 410 nm. Figure S5. (a) UV absorption spectra of different ratios of phospholipid-coated C1b (C1b⊂L) and transmission electron micrographs of the inclusions (b) Particle size test distribution of C1b⊂L. Figure S6. MCF-7 cell viability incubated with C1b⊂L measured by the CCK-8 assay. Results are presented as the mean ± SD in triplicate.
