# Supplementary material for: Photoacoustic Effect of Near-Infrared Absorbing Organic Molecules via Click Chemistry
Source: Molecules. 2022 Apr 4;27(7):2329. doi: 10.3390/molecules27072329 (PMC9000579; doi:10.3390/molecules27072329)
Supplement: Supplementary file 1 [file molecules-27-02329-s001.zip › molecules-1636331-supplementary.pdf]

Supporting Information

Photoacoustic Effect of Near-Infrared Absorbing Organic Molecules via Click Chemistry

Wenqing Zhu [1], Zongcheng Miao [1,*], Yaqin Chu [1], Liaoliao Li [2], Lei Wang [3,*], Dong Wang [2,*]

UV/Vis/NIR absorption of a series of NIR absorbing molecules: a series of near-infrared absorbing molecules were dissolved in dimethyl sulphoxide (DMSO) with the concentration of 3 × 10-5 M, respectively. The UV/Vis/NIR absorption spectra were obtained from Lambda 950, Perkin Elmer Instruments Co. Ltd with the wavelength ranges from 200 to 1400 nm, 1.0 nm slit width and absorption measurement mode).

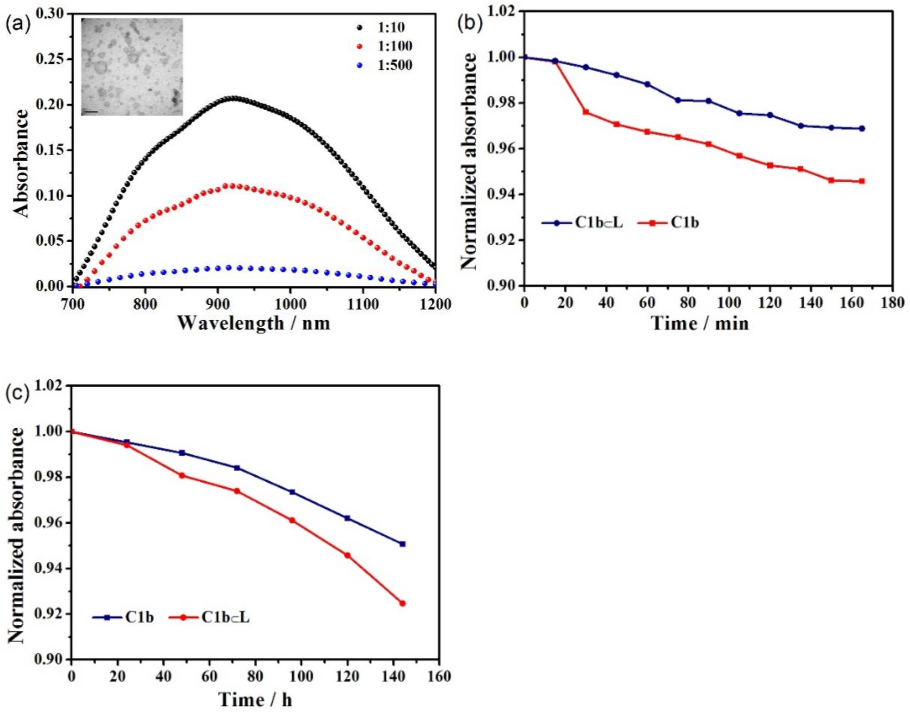

Figure S1. (a) UV/Vis/NIR absorption spectra of C1b⊂L with different ratios of C1b to liposomes in PBS and TEM images of C1b⊂L. (b) The UV/Vis/NIR absorption spectra were estimated after the solution of C1b and C1b ⊂L illuminating in every 15 min at 860 nm. (c) The UV/Vis/NIR absorption spectra were estimated after the solution of C1b and C1b ⊂L placing 24 h at 860 nm.

PA imaging in phantom: The C-XY solution was added into the agarose tube at a concentration of 3×10^{-5} mol/L. The phantom was scanned over the wavelength range of 680–940 nm through photoacoustic imaging by using MultiSpectral Optoacoustic Tomography (MOST) 128, which was purchased by iThera medical from Germany (Single-wavelength optoacoustic imaging at 10 Hz frame rate, Real-time spectral

component visualization at up to 5 Hz frame rate, Co-registered reflection-mode ultrasound computed tomography (R-UCT) imaging for MSOT inVision 512-echo, Penetration depth of 2–4 cm, sufficient for whole-body small animal imaging, Cross-sectional spatial in-plane resolution: 150 µm, High-energy/fast-tunable laser system (100 mJ/10 ms), Tomographic ultrasound detector array with 64/128/256/512 elements, Image acquisition fully automated). PA intensity measurements were obtained through mean pixel intensity of the same area of the images with the same intensity of laser pulse.

Thermal conversion efficiency: According to the previously reported methods, the value η can be calculated from the following equations:

$$\eta_{th}=\frac{mC_p(T_{\max}-T_{surrd})-Q_{Dis}}{\tau_s I(1-10^{-A(\lambda)})}$$

Equation S1

$$t=-\tau_s \ln\left(\frac{T-T_{surrd}}{T_{\max}-T_{surrd}}\right)$$

Equation S2

Where I was laser power, A(λ) was absorption of C-XY at 692, 842, 695, 866 nm, T_{max} and T_{surrd} were the temperature of maximum and surrounding, t was the time of cooling process after shutting off the laser, m was the mass of solution, C_p was the heat capacity, s was time constant, and Q_{Dis} could be ignored.

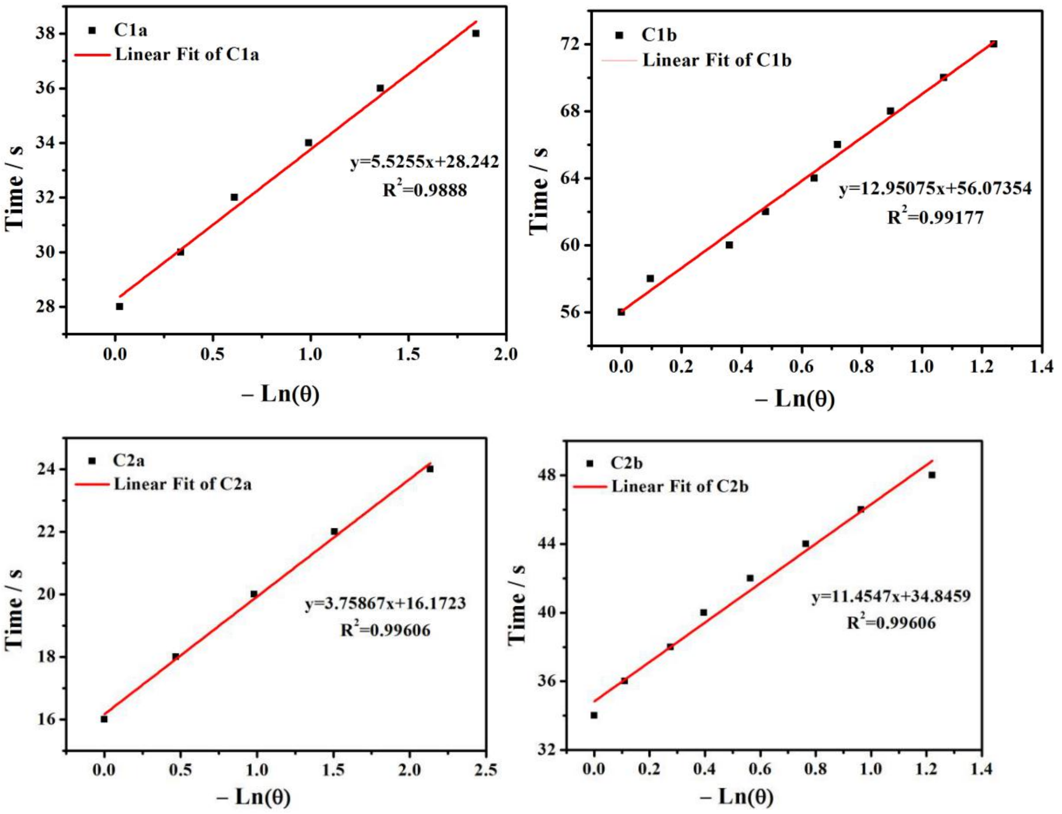

Figure S2. Time constants for heat transfer of C-XY were acquired by applying the linear time data from cooling period.

Detection of single line state oxygen:

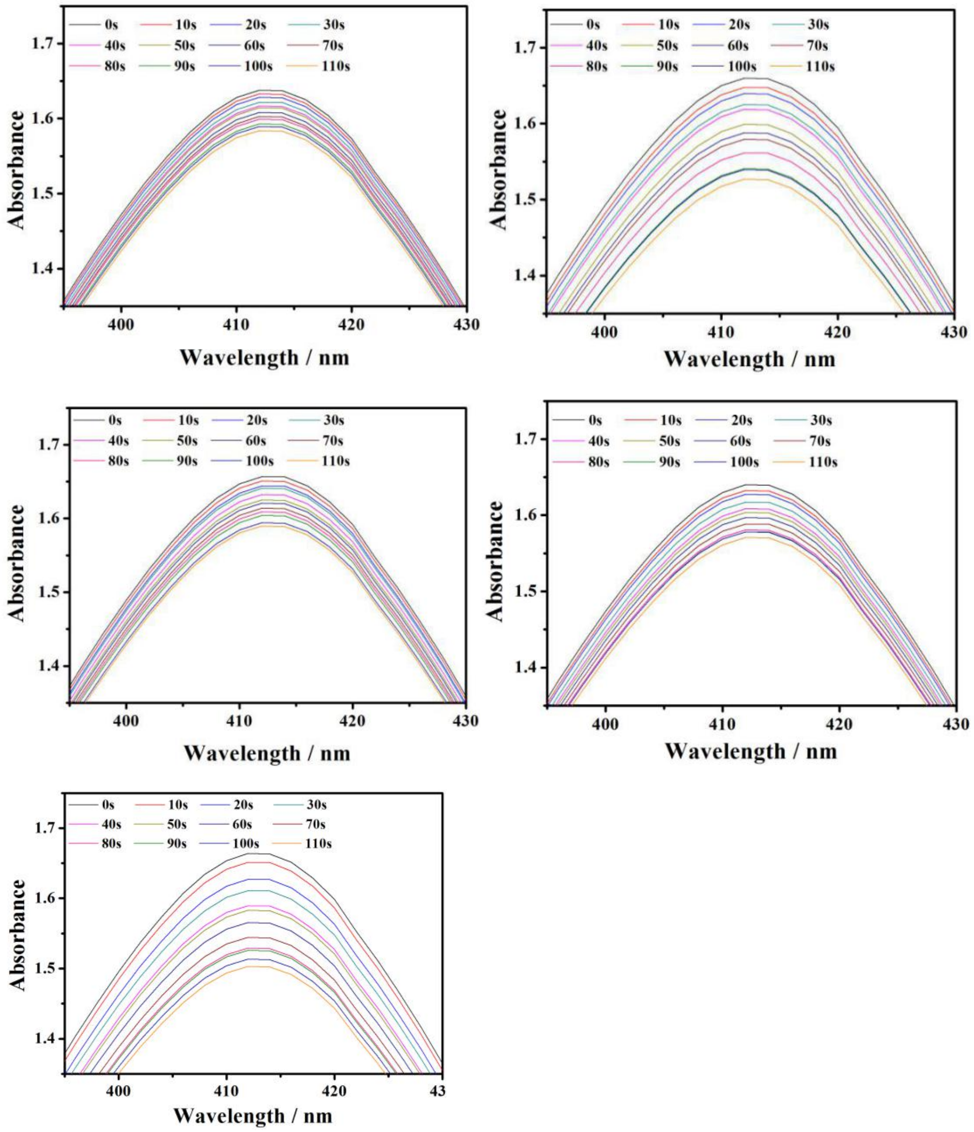

Figure S3. C1a, C1b, C2a, C2b, TPP in THF with DPBF for 10s at a time under a 650 nm light source

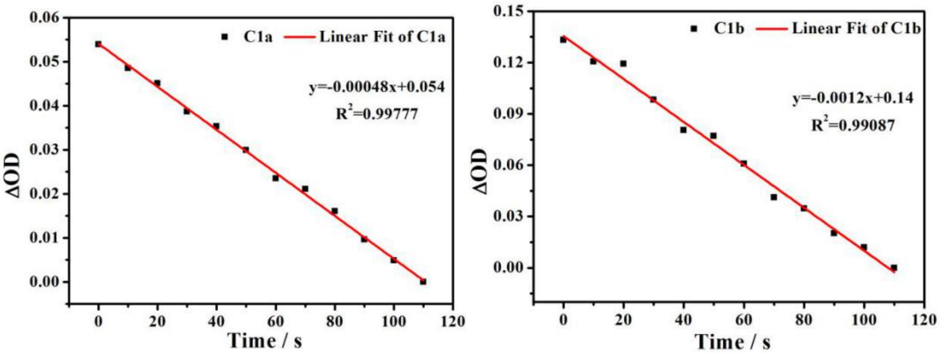

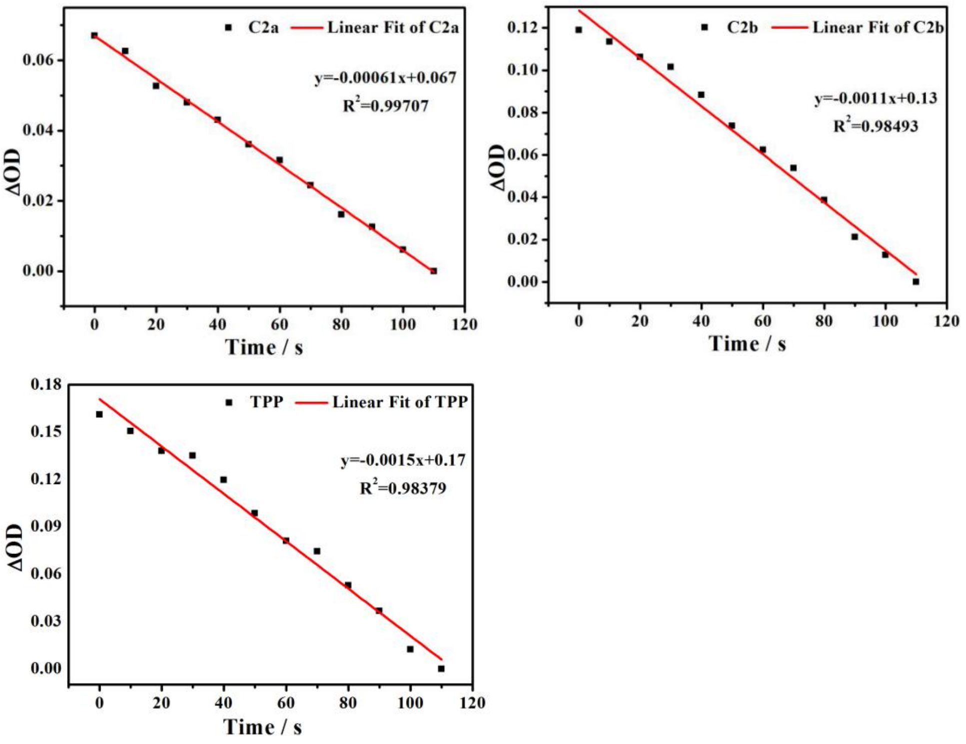

Figure S4. Linear fit of time and ΔOD at UV absorption of 410 nm

Phospholipid encapsulation of intermediate C1b:

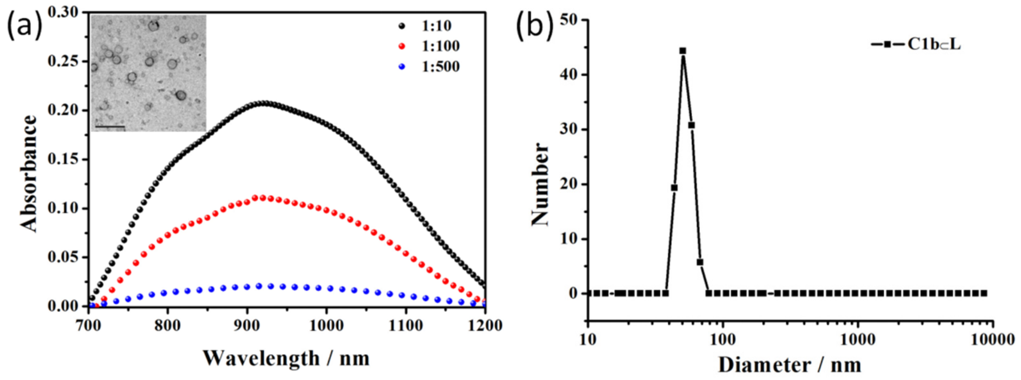

Figure S5. (a) UV absorption spectra of different ratios of phospholipid-coated C1b (C1b⊂L) and transmission electron micrographs of the inclusions (b) Particle size test distribution of C1b⊂L

Cytotoxicity Assay: MCF-7 cells (6×10^3 cells per well) at the logarithmic growth phase were incubated in 96-well culture plates for 17 h with DMEM containing 10% FBS and 1% penicillin (the whole mixed solution is 100 μL) at the temperature of 37 °C with 5% CO_2 atmosphere, and the culture medium was then replaced by fresh DMEM (100 μL). Afterwards, different concentrations of C1b⊂L were added into the 96-well culture plates filled with MCF-7 cells, respectively. After 24 h incubation at 37 °C in humidity and 5% CO_2 atmosphere, the mixture of solution in 96-well culture plates was removed and washed with PBS twice, and then 100 μL of fresh culture medium supplemented with 10% FBS and 1% penicillin were added into the wells. Subsequently, the solution of CCK-8 (10 μL) was added to the wells at 37 °C in humidity and 5% CO2 atmosphere.

Finally, the absorbance values of the cells per well were acquired by a Microplate Reader at 450 nm for analyzing cell viability through analyzing the fluorescence intensity of 96-well culture plates. Control experiments were done with addition of the same volume of PBS alone, and the other treatments were only added DMEM without adding C1b⊂L. The cell viability was calculated by the equation as follows: cell viability=(A_s-A_0)/A_c-A_0) ×100%. where the A_s was the absorbance of the different treatments with C1b⊂L, A_c was the absorbance of the control treatment with DMEM, A_0 was the absorbance of the control treatment with PBS.

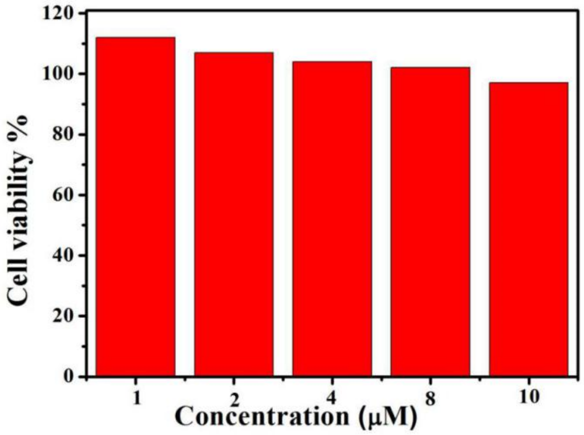

Figure S6. MCF-7 cell viability incubated with C1b⊂L measured by the CCK-8 assay. Results are presented as the mean ± SD in triplicate.
